# Supplementary material for: Nano Catalysis of Biofuels and Biochemicals from Cotinus coggygria Scop. Wood for Bio-Oil Raw Material
Source: Polymers (Basel). 2022 Oct 30;14(21):4610. doi: 10.3390/polym14214610 (PMC9659074; doi:10.3390/polym14214610)
Supplement: Supplementary file 1 [file polymers-14-04610-s001.zip › polymers-1946908-supplementary.pdf]

APPENDIX (Supplementary Information)

Table S1. GC-MS analysis of the extract of *Cotinus coggygria* Scop. wood.

No.	Retention Time (min)	Content (g /100 g)	Component
1	4.77	0.13	N,N-diethyl-Formamide
2	4.88	0.22	N,N-diethyl-Formamide
3	6.34	0.25	1-Hexanol, 2-ethyl-
4	10.76	0.02	Resorcinol
5	12.36	0.12	1,2,3-Benzenetriol
6	12.61	0.02	4-Chlorophenylhydrazine
7	15.44	0.03	5-methyl- [1,2,4]-Triazolo[1,5-a]-pyrimidin-7-ol
8	15.92	0.02	Levodopa
9	16.18	0.02	6-Hydroxybenzofuran-3-one
10	16.24	0.05	2-Naphthalenemethanol
11	16.59	0.27	2-Naphthalenemethanol
12	17.36	0.04	Aristol-1(10)-en-9-ol
13	18.75	0.03	(1R,4aR,7R,8aR)-7-(2-Hydroxypropan-2-yl)-1,4a-dimethyldecahydronaphthalen-1-ol
14	19.23	0.03	2-Naphthalenol, 2,3,4,4a,5,6,7-octahydro-1,4a-dimethyl-7-(2-hydroxy-1-methylethyl)
15	19.68	0.26	6-Isopropenyl-4,8a-dimethyl-1,2,3,5,6,7,8,8a-octahydro-naphthalen-2-ol
16	19.90	0.04	2-Naphthalenol, 2,3,4,4a,5,6,7-octahydro-1,4a-dimethyl-7-(2-hydroxy-1-methylethyl)
17	20.42	0.05	Estra-1,3,5(10)-trien-17.beta.-ol
18	20.49	0.02	1,2-Benzenedicarboxylic acid
19	20.70	0.06	4-(3,3-Dimethyl-but-1-ynyl)-4-hydroxy-3,5,5-trimethyl-cyclohex-2-enone
20	22.45	0.03	9,12-Octadecadienoic acid (Z,Z)-
21	22.50	0.03	Oleic Acid
22	26.48	0.04	.gamma.-Sitosterol
23	26.68	0.26	Homopterocarpin
24	26.92	0.04	Phenol, 4,4'-(1-methylethylidene)bis[2,6-dimethyl-
25	27.54	0.02	S-Indacene-1,7-dione, 2,3,5,6-tetrahydro-3,3,4,5,5,8-hexamethyl-
26	27.87	0.02	4H-1-Benzopyran-4-one, 2-(3,4-dimethoxyphenyl)-7-hydroxy-
27	28.07	0.02	10,11-Dihydro-10-hydroxy-2,3-dimethoxydibenz(b,f)oxepin
28	28.29	0.02	3(2H)-Benzofuranone, 6-methoxy-2-[(4-

			methoxyphenyl)-methylene]-, (E)-
29	28.40	0.01	Butanoic acid
30	28.77	0.01	10,11-Dihydro-2,3,6-trimethoxydibenz(b,f)oxepin-10-one
31	29.00	0.02	1H-2,8a-Methanocyclopenta[a]cyclopropa[e]cyclodecen-11-one, 5,6-bis(acetyloxy)-4-[(acetyloxy)methyl]-1a,2,5,5a,6,9,10,10a-octahydro-5a-hydroxy-1,1,7,9-tetramethyl-, [1aR-(1a.alpha.,2.alpha.,5.beta.,5a.beta.,6.beta.,8a.alpha.,9.alpha.,10a.alpha.)]-
32	30.22	0.05	Pregn-4-ene-3,20-dione, 16,17-epoxy-, (16.alpha.)-
33	30.67	0.02	4H-Cyclopropa[5',6']benz[1',2':7,8]azuleno[5,6-b]oxiren-4-one, 8,8a-bis(acetyloxy)-2a-[(acetyloxy)methyl]-1,1a,1b,1c,2a,3,3a,6a,6b,7,8,8a-dodecahydro-6b-hydroxy-3a-methoxy-1,1,5,7-tetramethyl-, [1aR-(1a.alpha.,1b.beta.,1c.alpha.,2a.alpha.,3a.alpha.,6a.alpha.,6b.alpha.,7.alpha.,8.beta.,8a.alpha.)]-
